# Dr. Reeves’ Biography

**Published:** 1892-01

**Authors:** 


DR. W. W. REEVES was a native of Virginia, having been born in Grayson county, June 23, 1847, and was thus only 44 years of age. He received his first education at the Jefferson High School, and afterwards read medicine with Dr. J. O. Wilcox, at Creston, Ash county, North Carolina. In 1874, he emigrated to Cedar Grove, Texas, where he lived until 1874, and then moved to Wills Point, at which place he resided until called to Austin by Governor Hogg to take charge of the lunatic asylum.

Dr. Reeves was twice married, the first time in 1873, when he married Miss Cora A. Horton. His second marriage occurred in 1879, his bride being Miss Margaret Knotzsch. He leaves five children, all of them being comparatively young.

Dr. Reeves came to Austin almost a stranger. By his gentle manners and courteous bearing,—by his many excellencies he won all hearts,—made a friend of all whom he met. The Board of Managers of the asylum passed most eloquent and touching resolutions on his death.

He was prominent in Masonic circles, being a Knight Templar, and a member of Ben Hur Lodge of the Mystic Shrine. A detachment of the Commandery K. T., of Austin, escorted the remains to Wills Point, the doctor's old home, and they were met at Hearne Junction by a similar detachment of K. T. from Dallas Commandery,—the encampment to which Dr. Reeves belonged. Governor Hogg also escorted the remains to Wills Point; and as a mark of respect to the memory of the deceased, the usual New Year's reception at the Governor's Mansion was dispensed with. The city of Austin was in mourning that day for a brave and good man, a popular and efficient officer, cut down in the prime of life, by the hand of one to whom he had been a benefactor.

